# Carisoprodol Abuse in Adolescence

**DOI:** 10.7759/cureus.11525

**Published:** 2020-11-17

**Authors:** Mayank Gupta

**Affiliations:** 1 Psychiatry, Clarion Psychiatric Center, Clarion, USA

**Keywords:** child and adolescent psychiatry, soma abuse, carisoprodol abuse, adolescent addiction, addiction psychiatry

## Abstract

Carisoprodol (i.e. Soma, Soprodol, Vanadom) is a muscle relaxant prescribed to relieve symptoms of muscle pain. Carisoprodol's addiction potential in adults has been well-established through case reports in the past. Carisoprodol abuse in adolescents has been reported in the ‘Monitoring the Future’ study since 2007, but no case studies or research has been published to date. Due to its relatively short half-life, tolerance and dependence develop quite quickly, leading to negative mental health outcomes. Awareness and education among health care providers remain critical to screen and treat this condition.

## Introduction

Carisoprodol (i.e. Soma, Soprodol, Vanadom) is a muscle relaxant prescribed to relieve symptoms of muscle pain. Carisoprodol was developed to promote a drug with less potential for abuse than meprobamate. However, numerous case reports have established carisoprodol as a drug of abuse [1]. It has barbiturate-like properties at the γ-aminobutyric acid type A (GABA-A) receptor, leading to central nervous system depression and reinforcing effects. The sought-out effects of carisoprodol are relaxation, giddiness, and drowsiness. It is typically consumed orally, however, when snorting the substance, the effects of euphoria appear much sooner, which may be the reason for that specific route of ingestion. When taken orally, carisoprodol converts to meprobamate in the liver. Carisoprodol starts to act within 30 minutes of ingestion; it has a half-life of eight hours. 1t is metabolized in the liver to hydroxy carisoprodol, hydroxy meprobamate, and meprobamate. Meprobamate is a Schedule IV drug, with the potential for abuse, and was widely prescribed in the 1970s to treat anxiety. The tolerance and antagonist-precipitated withdrawal of carisoprodol suggest that it has an addiction potential similar to that of another long-acting benzodiazepine [2]. This drug is typically abused orally by combining it with narcotic pain relievers and/or benzodiazepines. Studies suggest that when narcotic pain relievers, benzodiazepines, or alcohol are combined with carisoprodol, the effects of the drug are enhanced. When narcotic pain relievers and benzodiazepines are used together with the drug, an effect similar to heroin is created. The street terms for the drug include Ds, Dance, Las Vegas Cocktail (the combination of Soma and Vicodin), and Soma Coma (the combination of Soma and codeine). The side effects of carisoprodol are drowsiness and sedation when taken in excess. Due to the risk of physical and psychological dependence, the Food and Drug Administration (FDA) recommends only using carisoprodol for two to three weeks [3]. Patients that abuse carisoprodol may experience blurred vision, dizziness, drowsiness, and loss of coordination. The more severe side effects of carisoprodol abuse include chills, depression, tachycardia, tightness in the chest, vomiting, and unusual weakness. Patients dependent upon carisoprodol may go through withdrawal, which can include abdominal cramps, headache, insomnia, and nausea. An overdose may be indicated by difficulty breathing, shock, coma, and death.

## Case presentation

A 16-year-old Caucasian female was admitted after an intentional overdose of 50 sertraline (50 mg) tablets. Upon admission, the patient was guarded, agitated, inattentive, hyperverbal, and impulsive. She was experiencing feelings of helplessness, hopelessness, anhedonia, and had poor insight. The patient had a history of self-injurious behavior and had been previously hospitalized for unipolar depression and truancy. During her first hospitalization, the diagnosis of unipolar depression and generalized anxiety disorder was made and treated with escitalopram 10 mg daily. She later stopped the medication due to a lack of follow-up care. There were several psychosocial stressors affecting the patient, including the suicide of a close friend, loss of a job, traumatic injury to a close friend, and conflicts with mom. Her parents had divorced five years ago, and her primary caregiver was her mother. The patient was physically abused by her father who had a history of alcoholism and marijuana use. At age 14, the patient began drinking alcohol and using tobacco for recreational use. At the age of 16, she started snorting muscle relaxants, specifically carisoprodol, and occasionally used cannabis. She reported that both carisoprodol and cannabis helped her with anxiety. She denied using any other drugs. She initially started using one tablet (350 mg strength) of carisoprodol from her mother’s supply. She started to use carisoprodol due to easy access at home from her mothers' supply. She used it alone without any peers. After two weeks, it increased to two tablets and after a month, she was taking four tablets every day. Her attempts to stop abruptly resulted in withdrawal symptoms, which started within 36 hours. She eventually stopped its use a month prior to the said admission on the inpatient unit. She reported she was not able to get enough supply and self-tapered herself off it. Her vital signs at the time of admission were afebrile, pulse 80, blood pressure (BP) 125/80, and 100% saturation on air. The patient, based on a diagnostic assessment, met the criterion of bipolar 2 disorder and attention deficit hyperactivity disorder inattentive type in addition to her previous diagnosis of generalized anxiety disorder. She was treated with lithium 300 mg PO BID, quetiapine 100 mg HS, and sertraline 100 mg daily. She was also treated with methylphenidate ER 27 mg for her attention deficit hyperactivity disorder (ADHD) symptoms and, after a 10-day stay, was discharged from the hospital into the care of her mother. She engaged well with motivational interviewing-based psychosocial interventions. She agreed to follow up with the community-based multi-system treatment team [4].

## Discussion

Substance abuse and dependence have been extensively studied in adults, however, there is limited information on how abuse develops into dependency in adolescents. One in four older adolescents meet the criteria for abuse for at least one substance and one in five is found to meet the criteria for dependence on a substance [5]. Aarons et al. reported that 40.8% of adolescents in mental health settings met the criteria for lifetime substance use disorders and Lewinsohn et al. reported 60% of 14-18-year old with substance use disorders had another psychiatric disorder [6-7]. Patients diagnosed with adolescent-onset bipolar affective disorder (BPD) had a higher risk of developing substance use disorder as compared to patients with child-onset BPD, which was not preceded by conduct disorder or another psychopathology [8]. Childhood ADHD, oppositional defiant disorder (ODD), conduct disorder (CD), and depression increase the risk of developing substance-related disorders [9]. While the patient presented reported alcohol, tobacco, and marijuana use, she also reported nasal insufflation of muscle relaxants, specifically carisoprodol.

Carisoprodol (i.e. Soma, Soprodol, Vanadom) was not classified as a controlled substance initially, although it was frequently abused, and a psychological dependence developed. In January 2012, the Drug Enforcement Agency (DEA) classified carisoprodol as a Schedule IV controlled substance at the US federal level (Sch). The number of emergency room visits related to the misuse or abuse of carisoprodol increased from 15,830 visits in 2004 to 31,763 visits in 2009, with the number of patients aged 50 or older tripling during this time (2,070 to 7,115) and the number of patients aged 35-49 years old doubling (6,345 to 12,048), however, the number of younger patients did not significantly change. Seventy-seven percent of visits involved other pharmaceuticals; most commonly, narcotic pain relievers (55%) and benzodiazepines (47%). Thirty-five percent of ED visits required hospitalization from 2004-2009 [10]. According to the ‘Monitoring the Future’ study, the effects of carisoprodol are similar to those of tranquilizers like Xanax. While Xanax is the most widely abused tranquilizer, the prevalence of Soma abuse has gone down from 1.4 (2008) to 0.2 (2019). A recent report stated carisoprodol is prescribed more frequently to young adults for lower back pain [11].

Universal CRAFFT (Car, Relax, Alone, Forget, Friends, Trouble) screening is recommended for adolescents [12]. There are several evidence-based strategies that are effective in addressing these conditions. In primary care settings, screening, brief intervention, and referral to treatment (SBIRT) have demonstrated better outcomes [13]. The National Institute on Drug Abuse recommends the use of universal, selective, and indicated programs for prevention. The Life Skills Training (LST) Program, Adolescents Training and Learning to Avoid Steroids (ATLAS), and Project Towards No Drug Abuse (Project TND) are a few among many that have demonstrated effectiveness [14].

## Conclusions

Substance use disorders are common among adolescents with psychiatric disorders. Those with bipolar disorder that had an adolescent onset are at a greater risk of developing substance dependence as compared to those who experienced an earlier onset of the disease. There is one published case report in adults but it has not mentioned any co-morbid conditions. To my knowledge, no case reports of carisoprodol abuse in adolescents have been published. Carisoprodol abuse represents a major problem and scheduling it was one step in the right direction. However, its wide availability raises concern about its abuse potential and risks for other substance use. To decrease the risk of accidental abuse by children and adolescents, prescriptions for adults should be kept in a medicine safe or locked box to prevent easy access to others. There should be more focus on direct education to medical care providers to both screen and treat for other substances like carisoprodol.
